# Residual OXPHOS is required to drive primary and metastatic lung tumours in an orthotopic breast cancer model

**DOI:** 10.3389/fonc.2024.1362786

**Published:** 2024-05-01

**Authors:** Patries Herst, Georgia Carson, Danielle Lewthwaite, David Eccles, Alfonso Schmidt, Andrew Wilson, Carole Grasso, David O’Sullivan, Jiri Neuzil, Melanie McConnell, Michael Berridge

**Affiliations:** ^1^ Department of Cancer Cell Biology, Malaghan Institute of Medical Research, Wellington, New Zealand; ^2^ Department of Radiation Therapy, University of Otago, Wellington, New Zealand; ^3^ School of Biological Sciences , Victoria University of Wellington, Wellington, New Zealand; ^4^ Institute of Biotechnology of the Czech Academy of Sciences, Prague-West, Czechia; ^5^ School of Pharmacy and Medical Science, Griffith University, Southport, QLD, Australia

**Keywords:** breast cancer, orthotopic mouse model, metastasis, oxidative phosphorylation, glycolysis, intercellular mitochondrial transport

## Abstract

**Background:**

Fast adaptation of glycolytic and mitochondrial energy pathways to changes in the tumour microenvironment is a hallmark of cancer. Purely glycolytic ρ^0^ tumour cells do not form primary tumours unless they acquire healthy mitochondria from their micro-environment. Here we explored the effects of severely compromised respiration on the metastatic capability of 4T1 mouse breast cancer cells.

**Methods:**

4T1 cell lines with different levels of respiratory capacity were generated; the Seahorse extracellular flux analyser was used to evaluate oxygen consumption rates, fluorescent confocal microscopy to assess the number of SYBR gold-stained mitochondrial DNA nucleoids, and the presence of the ATP5B protein in the cytoplasm and fluorescent *in situ* nuclear hybridization was used to establish ploidy. MinION nanopore RNA sequence analysis was used to compare mitochondrial DNA transcription between cell lines. Orthotopic injection was used to determine the ability of cells to metastasize to the lungs of female Balb/c mice.

**Results:**

OXPHOS-deficient ATP5B-KO3.1 cells did not generate primary tumours. Severely OXPHOS compromised ρ^0^D5 cells generated both primary tumours and lung metastases. Cells generated from lung metastasis of both OXPHOS-competent and OXPHOS-compromised cells formed primary tumours but no metastases when re-injected into mice. OXPHOS-compromised cells significantly increased their mtDNA content, but this did not result in increased OXPHOS capacity, which was not due to decreased mtDNA transcription. Gene set enrichment analysis suggests that certain cells derived from lung metastases downregulate their epithelial-to-mesenchymal related pathways.

**Conclusion:**

In summary, OXPHOS is required for tumorigenesis in this orthotopic mouse breast cancer model but even very low levels of OXPHOS are sufficient to generate both primary tumours and lung metastases.

## Introduction

Despite significant progress in cancer treatments in the last two decades, metastatic disease remains responsible for more than 90% of cancer deaths. Understanding what drives the metastatic process in order to devise new anti-cancer strategies has been the topic of intense research (reviewed in (1)). Both intratumoral genetic heterogeneity and metabolic plasticity, in particular the ability to rapidly adapt energy-generating strategies to changes in the tumour microenvironment (TME), initiate and progress the metastatic cascade (2).

Both glycolysis and oxidative phosphorylation (OXPHOS) contribute to the total energy budget of all tumour cells. Although most cancer cells produce pyruvate required to fuel the TCA cycle during glycolysis, some cancer cells mainly generate pyruvate in the pentose phosphate pathway, which provides NADPH for biosynthetic pathways (3). Fatty acids and amino acids, such as glutamine, can also feed into the TCA cycle, as shown, for example, for triple negative breast cancer (TNBC) (4, 5). The contribution of OXPHOS to the total energy budget differs according to tumour cell type and physiological context. Differentiated non-malignant cells and slowly proliferating tumour stem cells derive most of their ATP from OXPHOS. However, rapidly proliferating cells such as less differentiated cancer cells and activated immune cells generate most of their ATP through aerobic glycolysis (6–8).

Metabolic plasticity, the ability to quickly adjust OXPHOS levels to fit the requirements of the changing TME, is now considered to be an established “hallmark of cancer” (9). Reports that describe changes in metabolic strategies between primary tumour cells, circulating tumour cells (CTCs) and metastatic cells vary considerably, depending on tumour type, TME composition, metastatic site and other parameters (reviewed in (7, 10)).

With respect to the metastatic ability of metabolically constrained cells, we have previously reported that purely glycolytic ρ^0^ cells do not generate primary tumours in mouse models of 4T1 breast cancer and B16 melanoma unless they acquire respiratory competent mitochondria from their host (11, 12). Intercellular mitochondrial transport completely restored respiratory capacity. Lack of *de novo* pyrimidine biosynthesis, rather than OXPHOS was shown to be responsible for the inability of ρ^0^ cells to generate primary tumours by themselves, due the absence of respiration, resulting in the loss of dihydroorotate dehydrogenase activity, an enzyme of the *de novo* pyrimidine biosynthesis pathway (12, 13) These ρ^0^ cells were also unable to seed in the lungs in a tail vein model (11).

In this manuscript, we test the hypothesis that an OXPHOS threshold is required to generate primary tumours and/or metastases. We used two 4T1 cell lines with severely compromised energy metabolism established previously (12). The ρ^0^D5 cell line, derived 5 days after sub-cutaneous injection of 4T1ρ^0^ cells into the flank of mice had very low OXPHOS, and the ATP5B-KO3 cell line lacked OXPHOS altogether. Although both cell lines had been shown to form primary tumours after subcutaneous injection (12), their ability to form lung metastases in an orthotopic mammary fat pad model in female Balb/c mice had not been explored and is the subject of this paper.

## Materials and methods

### Cell lines and cell culture techniques

The triple negative metastatic mouse cell line, 4T1, was obtained from the American Type Culture Collection. All cell lines were grown in full DMEM medium, which was supplemented with 10% (v/v) foetal bovine serum (ThermoFisher Scientific) and GlutaMAX-1 (2mM) and maintained at 37 °C and 5% CO_2_ in a humidified incubator. Penicillin (100 U/mL) and streptomycin sulphate (100 μg/mL) were added to *ex- vivo* cell cultures only. Full DMEM was further supplemented with uridine (50 μg/μL) and pyruvate (1 mM) for ρ^0^ cells. The ρ^0^ cells were derived previously by long-term culture (10-12 weeks) of 4T1 cells with 50 ng/mL ethidium bromide (11). Loss of mtDNA was monitored by the absence of the mitochondrial *Cytb* gene by PCR, and by pyruvate/uridine auxotrophy.

Because of the insensitivity of 4T1 cells to the cytostatic antimetabolite, 6-thioguanine (6TG), this drug is used to enumerate tumour cells from tissue. The ρ^0^D5 cell line was established previously from cells isolated in the presence of 60 µg (6TG), 5 days after subcutaneous injection of 10^6^ 4T1ρ^0^ cells into the flank of female Balb/C mice (12). The ρ^0^LM cell line was obtained previously from metastatic lung lesions in the presence of 60 µg 6TG after subcutaneous injection with 10^6^ 4T1ρ^0^ cells (11). The ρ^0^D5-LM cell line was generated from metastatic lung lesions in the presence of 60 µg 6TG after orthotopic injection of 10^6^ ρ^0^D5 cells. The ATP5B-KO3.1 cells were derived through serial dilution from ATP5B-KO3 cells, that were generated previously (12), using the CRISPR/CAS system (14).

## Materials

Unless otherwise noted, tissue plasticware was purchased from Nunc (ThermoFisher Scientific, Auckland, New Zealand); all cell culture reagents were from Gibco BRL (Invitrogen, Auckland, New Unless otherwise stated all other reagents were from Sigma Chemical Company (St. Louis, MO., U.S.A.).

### Measuring cell proliferation

The Incucyte S3 Live-Cell Analysis System (Sartorius) was used to measure confluency to evaluate cell proliferation rates and growth patterns of the 4T1, ρ^0^ and ρ^0^D5 cell lines. Cells were seeded in 6 well plates (2000 cells/well in full DMEM, with uridine and pyruvate supplementation for ρ^0^ cells only) and incubated at 37°C, 5% CO_2_ for 14 days, with media refreshed on day seven. Phase contrast scans (at 10x) were taken every 8 hours across 40 fields of view, analysed using Incucyte software and exported to excel spreadsheets to generate growth curves. Bright field images of individual fields of view were used to visualise growth patterns.

### Measuring oxygen consumption rates

The Seahorse XF96 extracellular flux analyser (Agilent) was used to measure cellular oxygen consumption rates (OCR) as per manufacturer’s instructions. Cells were seeded (30,000 per well) in Seahorse XF96 cell culture microplates pre-coated with poly-D-lysine solution (Agilent) the day before the experiment. After 15-18 h, the medium was replaced with Seahorse XF base medium supplemented with 5 mM HEPES, 10 mM glucose and 1 mM pyruvate with pH adjusted to 7.4, and plates were incubated without CO_2_ for 60 min. The Mito-stress assay protocol consists of four consecutive injection steps of 1 μM oligomycin, 2 μM FCCP, 0.5 μM rotenone/0.5 μM antimycin A and lastly 5 μg/ml Hoechst 33342 to determine cellular abundance. Data were normalized to Hoechst staining intensity (absorption at A490, Tecan Plate reader). Raw datasets were exported to excel to generate oxygen consumption profiles.

### Mouse experiments

Balb/c mice were housed in the Biomedical Research Unit at the Malaghan Institute of Medical Research. Female mice between six and ten weeks of age were used for intravenous (IV) or orthotopic (mammary fat pad) injection of tumour cells. All animal experimentation was carried out under animal ethics approval from Victoria University of Wellington (#29071). Lungs were perfused with 20mL PBS before removal; the left lobe was embedded and stained, the right lobes were used to generate metastases and to derive cell lines. For the orthotopic metastasis model, 10^6^ tumour cells in 50 μL PBS were injected into the fourth mammary fat pad of female Balb/c mice using a 25 mL gauge needle. Mice were culled via CO_2_ to avoid damaging the arteries needed for perfusion, when a tumour reached 0.5cm^3^ or ulcerated, or if mice lost 10% bodyweight in 24h or were otherwise unwell. Lungs were excised and used in metastases assays. Mice that did not develop primary tumours were culled at 60 days and tissue around the injection site was macerated and incubated in the presence of 60 µg 6TG in T75 flasks to check for the presence of latent tumour cells. For the IV injection experimental metastasis model, 10^6^ tumour cells in 50 μL PBS were injected into the lateral tail vein using a 25 mL gauge needle. Animals were culled using a CO_2_ chamber 3 weeks later.

### Presence of lung metastases

Lung sections were macerated in a petri dish, type 4 collagenase (1 mg/mL in DMEM) was added, and samples were incubated in a shaking incubator (88 rpm, 37°C for 3 h). Digested tissue was strained and centrifuged. Cell pellets were resuspended in DMEM in the presence of 60 µg 6TG in T75 flasks. Medium was replaced after 7 days and flasks were scored for the presence or absence of colonies 7 days later.

### Haematoxylin & eosin staining

Lung sections for use in tumour histology were perfused with 20mL PBS before being incubated in 4% PFA for 24 hours at 4°C. Samples were dehydrated with a series of EtOH incubations, cleared with xylene, embedded in paraffin wax and sectioned on a Microm HM 325 Rotary Microtome at a thickness of 5 μm, and mounted on slides. FFPE samples were dewaxed, dehydrated and stained with haematoxylin (2.5 min) and eosin (1 min), washed, dehydrated, cleared and cover-slipped. H&E sections were imaged on the VS200 Slide Scanner (Olympus) using brightfield settings. OIR files were visualised using QuPath.

### Presence of ATP5B protein in cell lines

Cells were plated in an 8-well chamber slide at 3 x 10^4^ cells/well and incubated at 37°C overnight. Cells were washed (3x, PBS), fixed (4% PFA, 15 min at RT), washed (3x, PBS) and permeabilised (0.1% Triton X-100, PBS, 15 min on ice), washed, blocked (5% BSA in PBS; 1 h on ice) and incubated with anti-ATP5B-AF488, clone 4.3E8.D10 (Invitrogen MA1-930-A488, LOT WF330749) (1.25 µg/mL) in 0.1% Triton X-100 + 1% BSA in PBS (1 h at RT) and washed and stained with 10 ng/mL DAPI (in PBS) or nucGreen (10 min at RT). Images were acquired as z-stacks across the focus depth, in an Olympus inverted microscope IX83 equipped with Laser Scanning Confocal Microscope head (FV3000) with a 405nm (50 mW), 514 nm (40 mW), 516 nm excitation lasers and sensitive detectors with emission bands of 430-470, 530-580 and 610-respectively. Confocal image files were pre-processed using a FIJI macro to compress z-stacks (15). CellProfiler pipelines were generated to quantify cell ATP5B staining (16).

### Presence and abundance of mtDNA in cell lines

Cells were plated in an 8-well chamber slide as above. Cells were washed (3x PBS) and incubated (45 min at RT) in 300 mL staining mixture, consisting of SYBR gold (1:40,000), MitoTracker CMXRos (1: 20,000; 50nM) and Hoechst 33342 (ThermoFisher Scientific) (1:10,000). Images were acquired as above. Maximum Intensity projection images were segmented by nuclei (Hoechst), cytoplasm (MitoTracker CMX-ROS) and mitochondrial DNA fluorescent signals (SYBR Gold). Each mtDNA nucleoid was counted and allocated to its respective cytoplasm segmentation excluding the nuclear area (see **Supplementary Figure 2**). Images were processed and analysed using FIJI software (15) and CellProfiler (16). A three-dimensional reconstruction was made using IMARIS (Bitplane, v 9.8.0).

### MinION cDNA preparation, sequencing, mapping and analysis preparation

RNA from the different 4T1 cell lines was extracted (Qiagen RNAeasy Mini Kit) and quantified using the QuantiFluor^®^ RNA System (Promega). RNA (500 ng) was used for library preparation for Oxford Nanopore Technologies (ONT) long-read cDNA sequencing. Only cDNA greater than 20 ng/uL was used in sequencing libraries. Briefly, this process involves reverse transcription of extracted RNA using a custom poly TVN primer that binds to polyA RNA sequences; second-strand synthesis using ONT-provided strand-switch primer, followed by PCR to incorporate barcoded ONT rapid attachment primers, then binding of rapid adapters. Samples preparation varied between early R9.4.1 runs and later R10.4.1 runs (in August and September 2023). Early samples from R9.4.1 runs were processed according to the latest ONT cDNA protocol available at the time of sequencing (e.g SQK-PCS108 with SQK-PBK004, SQK-PCB109). Up to 6 samples were multiplexed together in equimolar quantities for each run (with technical duplicate PCR reactions for some samples), then run on an ONT MinION using an R9.4.1 MinION flow cell. Later samples from the R10.4.1 runs were processed according to an in-house developed protocol that combines the ONT SQK-PCB111 PCR-cDNA barcoding kit with Kit14 rapid adapter, to allow the sequencing to be carried out on R10.4.1 flow cells. Up to 14 samples were multiplexed together in equimolar quantities for each run (with technical duplicate PCR reactions for some samples), then run on an ONT P2 Solo using an R10.4.1 PromethION flow cell. Our current protocol for this is attached.

### MinION data analysis

The program LAST was used to identify ONT barcodes present in sequenced reads, followed by a customised program (17), designed to use barcode assignments to demultiplex reads into files based on their incorporated barcodes. Demultiplexed reads were then mapped to the ONT strand switch primer sequence in order to identify the direction of transcription. Reads were mapped to the mouse transcriptome using LAST, grouped by mapped transcript, and counted producing a table that was further processed using DESeq2 (18, 19) to determine differential expression (log_2_ fold change) of ρ^0^SC vs ρ^0^LM and ρ^0^D5 vs ρ^0^D5LM. We used FDR-adjusted *p* values reported by DESeq2 (20) of 0.1 as a threshold for statistical significance. Whole-transcriptome processing differed between early R9.4.1and later R10.4.1 runs. For all R9.4.1. runs, reads were base-called with guppy v5.1.15-v6.4.8 using the most recent version of the generic DNA model at the time of calling in super-accuracy mode (dna_r9.4.1_450bps_sup). Older runs were recalled using this newest model to maintain calling consistency. For all R10.4.1 runs, reads were base-called with Dorado v0.4.0 + 0aaf16d using the most recent version of the generic DNA model at the time of calling in super-accuracy mode (dna_r10.4.1_e8.2_400bps_sup@v4.2.0). Reads were then demultiplexed using LAST [https://dx.doi.org/10.17504/protocols.io.14egnxw4zl5d/v8], oriented so that the sequence direction matched the transcription direction [https://dx.doi.org/10.17504/protocols.io.5qpvon2zzl4o/v7], then mapped to an A/T homopolymer-masked version of the Gencode M28 mouse transcriptome using LAST and converted into transcript count tables [https://dx.doi.org/10.17504/protocols.io.5qpvonn2bl4o/v17].

### Differential expression analysis

Count tables were processed using DESeq2 v1.40.2, using a statistical model that incorporated cell lines and sequencing runs as independent factors (i.e. “design = ~ line + run”). Differentially-expressed log fold-change values were shrunk using the apeglm method [10.1093/bioinformatics/bty895], including the shrunk adjusted p-value calculation. Variance-stabilised values (used for expression plots) were generated using the ‘vst’ function of DESeq2, and linearly scaled to set the minimum log expression level to 0 while maintaining the 99th percentile expression level.

### GSEA analysis

Shrunk log fold change values were used as input in the ‘fgsea’ function of fgsea v1.26.0, splitting and separately calculating enrichment for upregulated and downregulated genes, matching against mouse gene sets H, C1, C2, and C3 from the ‘msigdb’ package. All hallmark gene sets were summarised, showing normalised enrichment scores and unadjusted *p*-values produced in the fgsea results.

### Cytogenetic culture harvesting

4T1 cells were fixed and processed according to methods published in protocol 4.12 of the Association of Genetic Technologists (AGT) cytogenetics manual (21). Fixed 4T1 cytogenetic cell suspensions were fixed and slides were dehydrated in a 70%, 80%, and 95% ethanol series (3 min each). Following air drying, 1 μL of mouse anti- ATP5B/Cen10 FISH probe (Empire Genomics, USA) was resuspended in 4 μL hybridization buffer (Empire Genomics) and pipetted onto a 22x22 mm glass coverslip and co-denatured at 75°C for 4 min, incubated overnight at 37°C in a humidified chamber to permit hybridization of the probe to the target DNA. Following post-hybridization washes, ProLong™ Diamond Antifade mountant with DAPI (Thermo Fisher Scientific) was added. Slides were analysed using an Olympus VS200 slide scanner for fluorescent image analysis using a 60x oil objective. Nuclei on each FISH slide were analysed for both ATP5B and Cen10 probe signals.

## Results

### Growth and oxygen consumption profiles of respiration-compromised 4T1-derived cell lines

**Figure 1:**
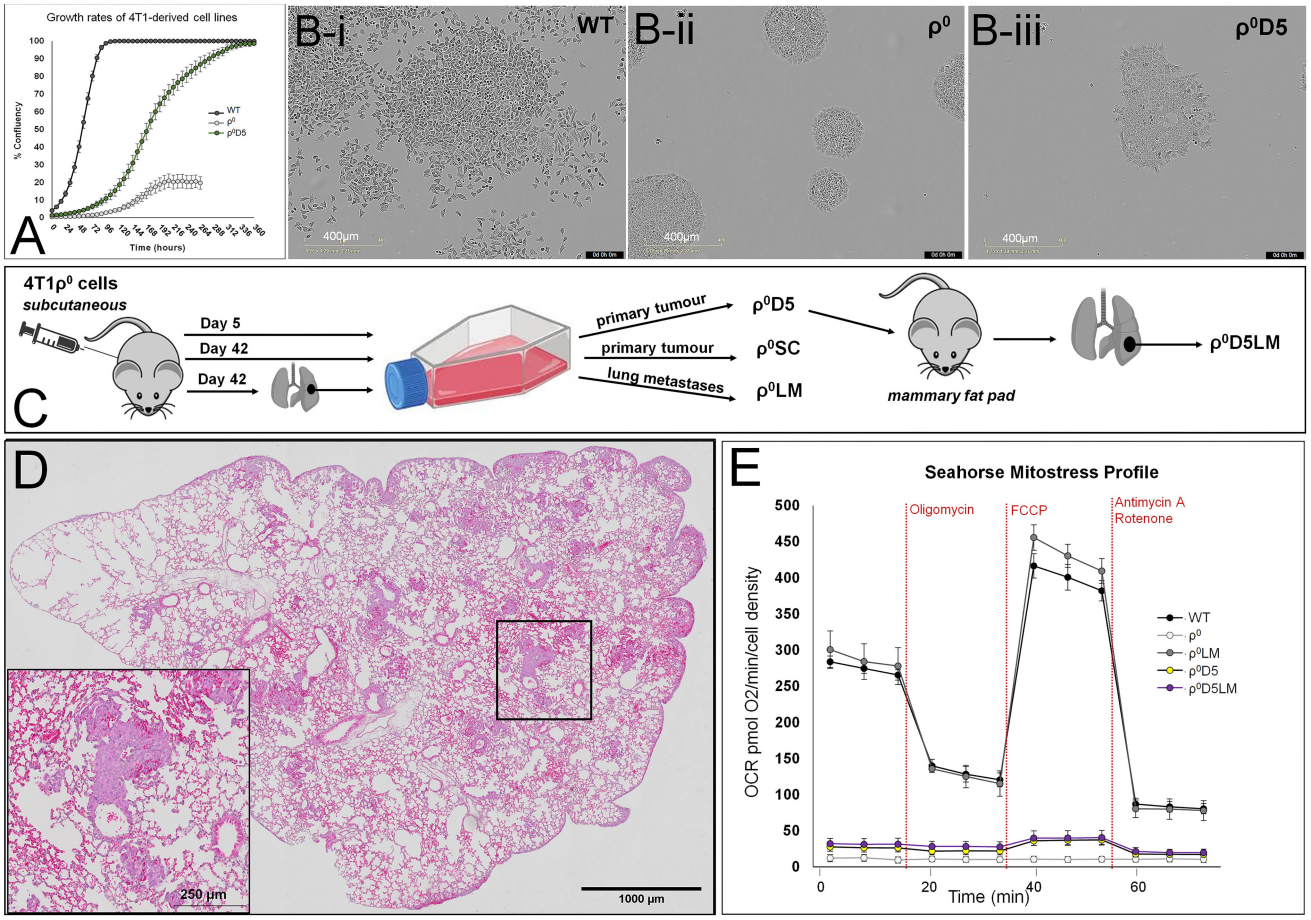
Growth and oxygen consumption profiles of 4T1 cell lines. **(A)** Growth rates as % confluency of WT, ρ^0^ and ρ^0^D5 cells over 2 weeks after seeding at 2000 cells per well in 6 well plates, using the Incucyte S3 Live-Cell Analysis System; **(B)** Typical growth patterns of these cell lines at similar confluency levels; **(C)** Diagrammatic representation of 4T1-derived cell lines; **(D)** Representative H&E image of a lung showing metastatic lesions. Insert is a magnification of the square in the overview image, clearly showing tumour cells forming small metastases in the lung. **(E)** Mitostress Seahorse profiles of 4T1-derived cell lines. Oligomycin inhibits respiratory complex V, stopping ATP-linked OCR (OXPHOS). FCCP dissipates the mitochondrial membrane potential, increasing OCR to maximal respiratory capacity. Rotenone/Antimycin A block respiratory complex I/III respectively, stopping all mitochondrial oxygen consumption. Incucyte experiment data are based on three biological replicates, Seahorse data are an average and SEM of at least 4 biological replicates.

A comparison of growth rates (**Figure 1A**) and growth patterns (**Figure 1B**i-iii) of the ρ^0^D5 cells with ρ^0^ and WT cells showed that ρ^0^D5 cells grew slower and in a clumpy growth pattern compared to WT cells; ρ^0^ cells grew very slowly in tight clusters and only reached 20% confluency. When injected into the mammary fat pads of female Balb/c mice, ρ^0^D5 cells generated both primary tumours and lung metastases (**Figures 1C, D**, **Table 1**).

**Table 1:** Summary of metabolic and metastatic capacity of different 4T1 cell lines.

4T1 Cell lines	Basal OCR * ave ± SEM (%WT)	OXPHOS * ave ± SEM (%WT)	Glycolysis ** ave ± SEM (%WT)	Primaries	Lung metastases
WT	195 ± 7.3 (100%)	149 ± 9.7 (100%)	42 ± 1.27 (100%)	15/15 ^#^	15/15 ^#^
ρ^0^	1 ± 4.7 (0%)	0 ± 3.7 (0%)	75 ± 12.2 (179%)	15/15 after mitotransfer ^#^	15/15 after mitotransfer ^#^
ρ^0^LM	208 ± 25.3 (107%)	162 ± 10.6 (109%)	45 ± 1.91 (107%)	13/14 ^#^	0/4
ρ^0^D5	10 ± 5.8 (5%)	45 ± 3 (3%)	50 ± 4.42 (119%)	15/19	15/19
ρ^0^D5-LM	12 ± 7.7 (6%)	30 ± 2 (2%)	23 ± 1.8 (55%)	13/14	2/14
ATP5B-KO3.1	11 ± 2 (6%)	0 ± 0.2 (0%)	40 ± 2.46 (95%)	1/18 ^##^	0/18

* OCR = pmol O2/min; ** ECAR = mpH/min; #: Tan et al., 2015 or combined with Tan et al, 2015; ##: primary tumor was formed by ATP5B generating tumor cells.

We hypothesized that ρ^0^D5 cells would improve their respiratory capacity by obtaining additional healthy mitochondria from the mouse, similar to ρ^0^ cells. We used the Mitostress protocol of the Seahorse extracellular flux analyser to evaluate oxygen consumption rates (OCR) before and after exposing cells to the respiratory complex V inhibitor, oligomycin (**Figure 1E**). ATP-linked OCR can be considered a measure of OXPHOS. Comparing Seahorse profiles, basal OCR and ATP-linked OCR of ρ^0^ and ρ^0^D5 cells with those of the corresponding metastatic lung cells, we confirmed previous findings that basal OCR and ATP-linked OCR of ρ^0^LM cells closely resembled that of WT cells (p=0.340 and 0.235 respectively; t-test) (**Figure 1E**). However, contrary to expectation, the basal and ATP-linked OCR of ρ^0^D5LM cells isolated from lung lesions remained similar to those of ρ^0^D5 cells (p=0.363 and 0.300 respectively; t-test) (**Figure 1E**).

In summary, WT and ρ^0^LM had robust OXPHOS levels but both ρ^0^D5 and ρ^0^D5LM cells had very poor OXPHOS levels.

### Oxygen consumption profiles of OXPHOS^null^ ATP5B-KO cells

Previous research with ATP5B KO3 and KO7 cells showed that these cells had no OXPHOS, yet generated primary tumours (12). We initially confirmed these findings for ATP5B-KO3 cells. However, we found that this cell line sporadically expressed ATP5B protein in 1-2% of cells (**Supplementary Figure 1A**). This small number of ATP5B-expressing cells expanded rapidly over time (**Supplementary Figure 1B**). ATP5B expression correlated to increased ATP-linked OCR (**Supplementary Figure 1C**). Although ATP synthase activity was completely inhibited by oligomycin, this activity was re-established soon after oligomycin was removed from the medium (**Supplementary Figure 1D**) and therefore this approach was unsuitable for *in vivo* experiments.

**Figure 2:**
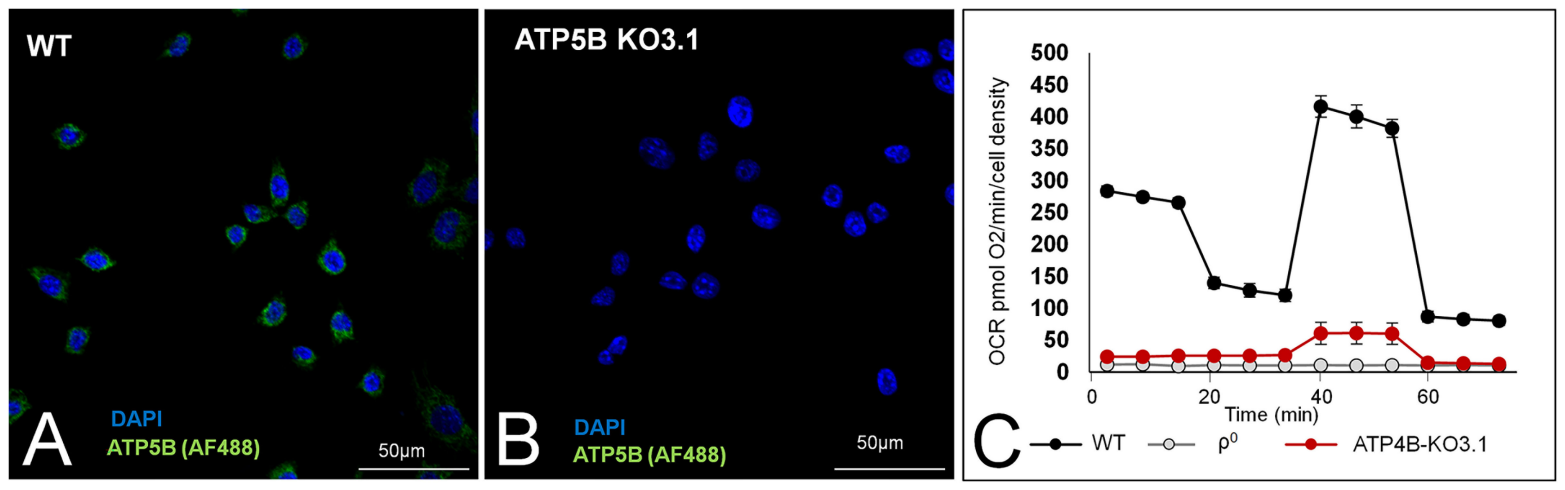
ATP5B expression and respiration of WT, and ATP5B-KO3.1 cells. Representative images of ATP5B staining in WT cells **(A)** and ATP5B-KO3.1 cells **(B)**; **(C)** Mitostress Seahorse profile of WT, ρ^0^ and ATP5B-KO3.1 cells, showing a complete lack of OXPHOS in both 4T1ρ^0^ and ATP5B-KO3.1 cells. Seahorse data are an average and SEM of at least 4 biological replicates.

We removed ATP5B-expressing cells from ATP5B KO3 cells by means of serial dilution of an early culture and found that ATP5B KO3.1 cells did not express the ATP5B protein over a 12-month period. Representative images of the presence/absence of the ATP5B protein in WT cells and ATP5B KO3.1 cells are shown in **Figures 2A, B**, respectively. **Figure 2C** shows low basal OCR of ATP5B KO3.1 cells (5% of 4T1 cells), similar to that of ρ^0^D5 cells (p= 0.721; t-test) and ρ^0^D5LM cells (p=0.173, t-test). In addition, ATP5B KO3.1 cells had no ATP-linked OCR (**Figure 2C**). The clonal status of ATP5B KO cell lines was explored by nuclear *in situ* hybridization using ATP5B/Cen10 FISH probes, showing that WT, ATP5-KO3 and ATP5B-KO7 cells contained both diploid and triploid cells, but ATP5B-KO3.1 cells were exclusively triploid (**Supplementary Figures 1D, E**).

In summary, we showed that ATP5B-KO3.1 cells could not perform OXPHOS.

### Linking energy profiles to metastatic ability

**Figure 3:**
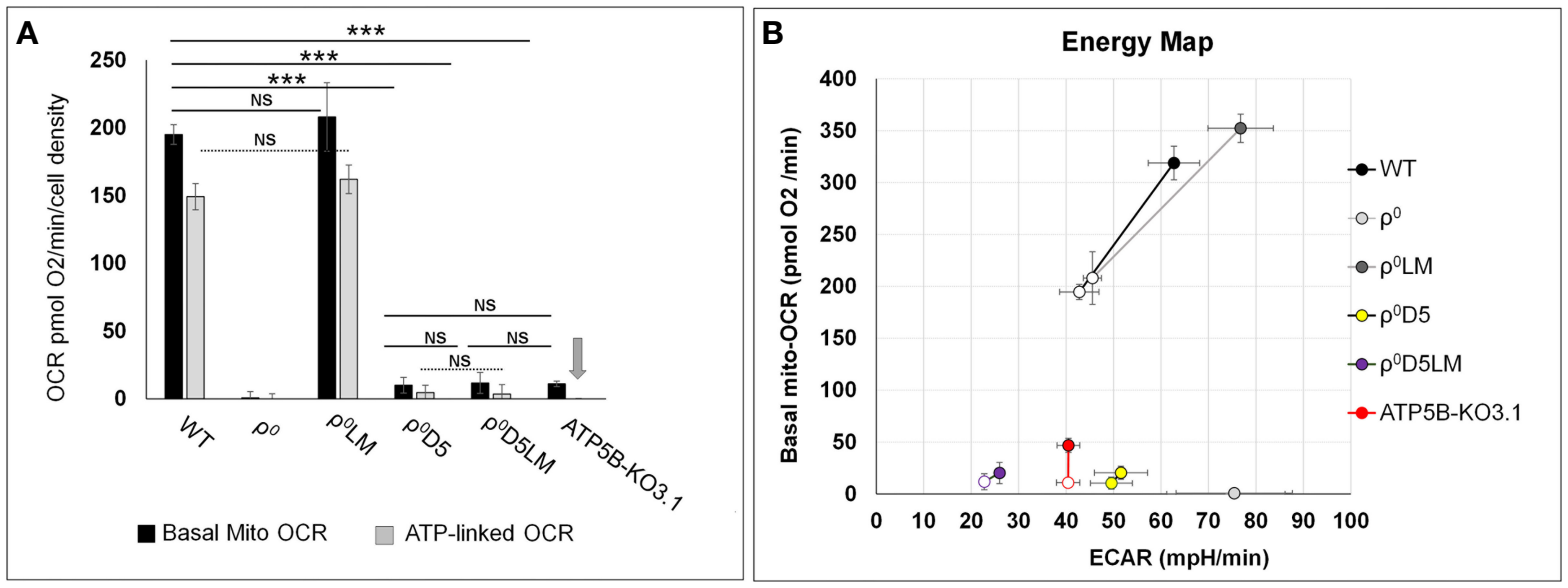
Comparison of energy metabolism parameters between 4T1-derived cell lines. **(A)** Bar graphs depicting basal and ATP-linked mitochondrial OCR of 4T1 and derived cell lines. Arrow denotes lack of ATP-linked OCR in ATP5B-KO3.1 cells; **(B)** Energy maps of unstressed (open symbols) and stressed (closed symbols) OCR and ECAR of 4T1 and -derived cell lines. Stressed OCR were measured after exposure to FCCP and stressed ECAR after exposure to oligomycin. The stress response is a measure of adaptability to different microenvironment stressor. Statistically significance using the t-test: ***= p<0.001. NS= not statistically significant (p>0.05). Seahorse data are an average and SEM of at least 4 biological replicates.

Differences between basal OCR of ρ^0^D5/ρ^0^D5LM, ρ^0^D5/ATP5B-KO3.1 and ρ^0^D5LM/ATP5B-KO3.1 cells were not significant (p=0.363, 0.721 and 0.173, respectively; t-test) (**Figure 3A**). Energy profiles of the 4T1 cell lines shown in **Figure 3B** were generated by plotting basal OCR (mito-OCR) against extracellular acidification rates (ECAR) under unstressed and stressed conditions. The increase in OCR after exposure to the ionophore FCCP, which dissipates the proton gradient, is often referred to as the respiratory reserve, and is an indication of maximum OCR. The increase in ECAR after oligomycin addition can be referred to as glycolytic reserve, which occurs because blocking OCR will result in attaining maximum levels of glycolytic rates.

With respect to metastatic capacity, ATP5B-KO3.1 cells were unable to generate primary tumours when injected orthotopically in female Balb/c mice (n=17/18) (**Table 1**). Tumour cells of the one mouse that did develop a primary tumour, expressed the ATP5B protein (**Supplementary Figure 1F**). The ρ^0^D5 cell line generated primary tumours and lung metastases in 15 out of 19 mice whereas ρ^0^D5LM cells from 4 separate clones formed primary tumours in 13 out of 14 mice in 4 separate experiments, but only 2 out of the 14 mice developed lung metastases (**Table 1**). In order to further explore the ability of cell lines generated from lung lesions to form lung metastasis themselves, we orthotopically injected a small number of mice (n=5) with fully respiratory-competent ρ^0^LM cells (11). These cells were previously shown to form primary tumours (in 10/10 mice), and in our experiments, 4 out of 5 mice developed primary tumours. However, none of these 4 mice developed lung metastases. Using a different model of lung seeding, whereby tumour cells are directly injected into the tail vein, we found that ρ^0^D5LM cells did form lung nodules (n=7/7) in this model, unlike ATP5B-KO3.1 cells (n=0/5).

In summary, metastatic ability does not appear to be linked to either OCR, OXPHOS or respiratory reserves in our cell lines. Both WT and ρ^0^SC cells with robust OCR, OXPHOS and stress responses as well as ρ^0^D5 cells with very poor OCR, OXPHOS and stress responses all consistently generated metastases. In contrast, poorly performing ρ^0^D5LM cells and highly performing ρ^0^LM cells only generated metastases in very few of the mice. ATP5B-KO3.1 cells with no OXPHOS did not generate primary tumours.

**Figure 4:**
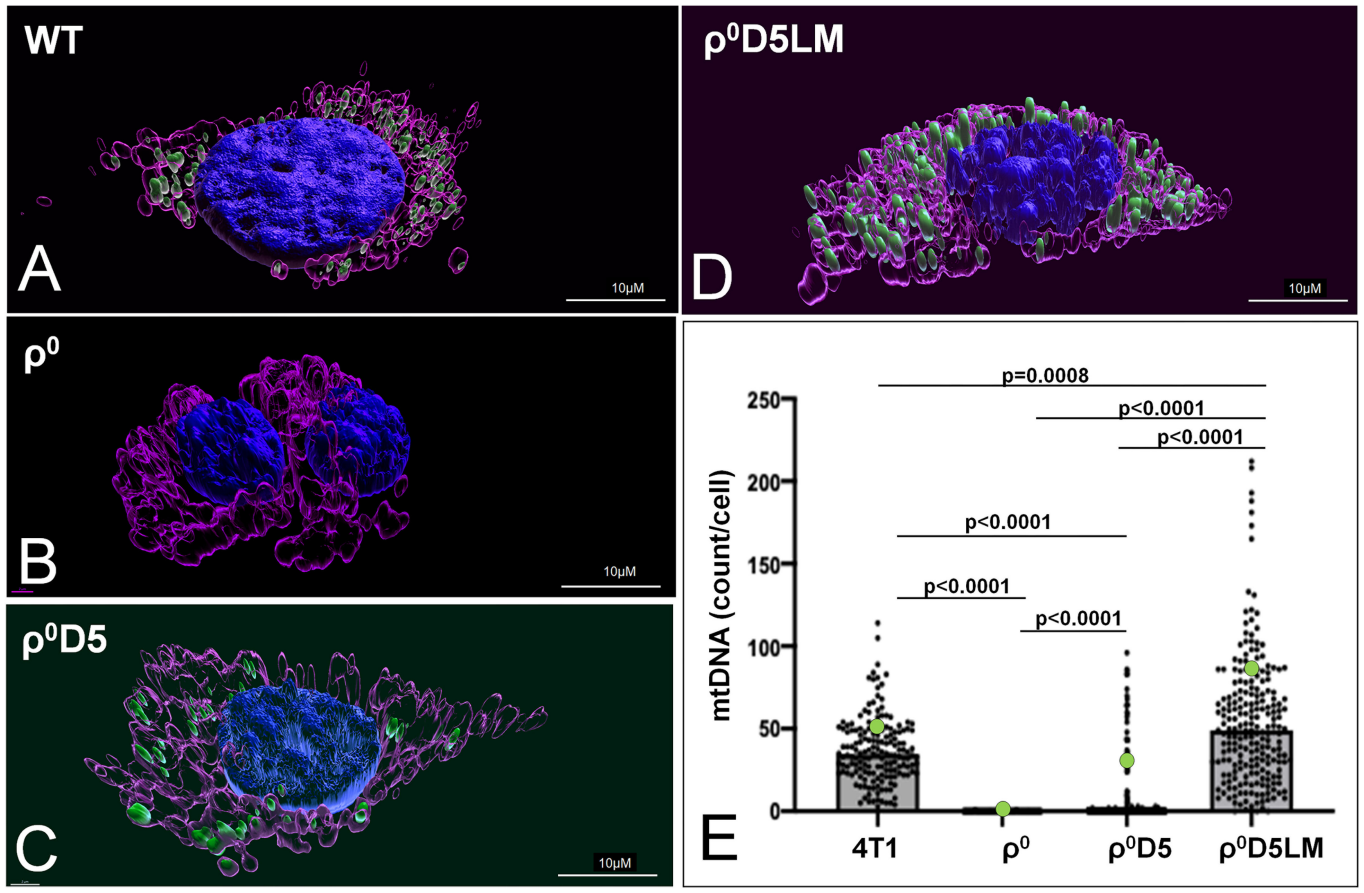
Mitochondrial DNA abundance in 4T1-derived cell lines. **(A–C)** Live images of 4T1 cells stained with MitoTracker CMXROS (magenta), SYBR Gold (green) and an overlay of those two together with Hoechst (blue). **(A–D)** 3-Dimensional images of live cells of 4T1 cells stained using IMARIS version 9.8.0. **(E)** Number of mtDNA nucleoids per cell of different 4T1 cell types, using an imaging pipeline shown in S**upplementary Figure 2**. Nucleoids were counted in 166 WT cells, 212 ρ^0^ cells, 347 ρ^0^D5 cell and 218 ρ^0^D5LM cells. Statistical significance was determined using the non-parametric Kruskall-Wallis test with Dunn’s multiple comparisons test as the data for these data sets were not normally distributed.

Because of the failure of ρ^0^D5LM cells to improve their respiratory capacity, we explored whether or not these cells were unable to acquire respiration-competent mitochondria, in contrast to ρ^0^ cells (11). We assessed the number of mtDNA nucleoids in individual cells using the mtDNA-specific dye SYBR Gold (22) to stain mtDNA nucleoids, MitoTracker CMXROS (23) to stain mitochondria and Hoechst 33342 to stain nuclei. Representative three dimensional renderings of WT, r0, r0D5 and r0D5LM are shown in **Figures 4A–D**. An Image analysis pipeline analysed cells based on their mitochondrial content (MitoTracker CMXROS-positive area). The SYBR Gold signal located underneath or on top of the nucleus was subtracted and the number of mtDNA nucleoids per cell calculated and tabulated (**Supplementary Figures 2A-F**). The results are presented in **Figure 4E** and validate previous findings that only 10-15% of ρ^0^D5 cells contain mtDNA (12).

In summary, low respiratory capacity was not linked to low mitochondrial DNA (mtDNA) abundance.

**Figure 5:**
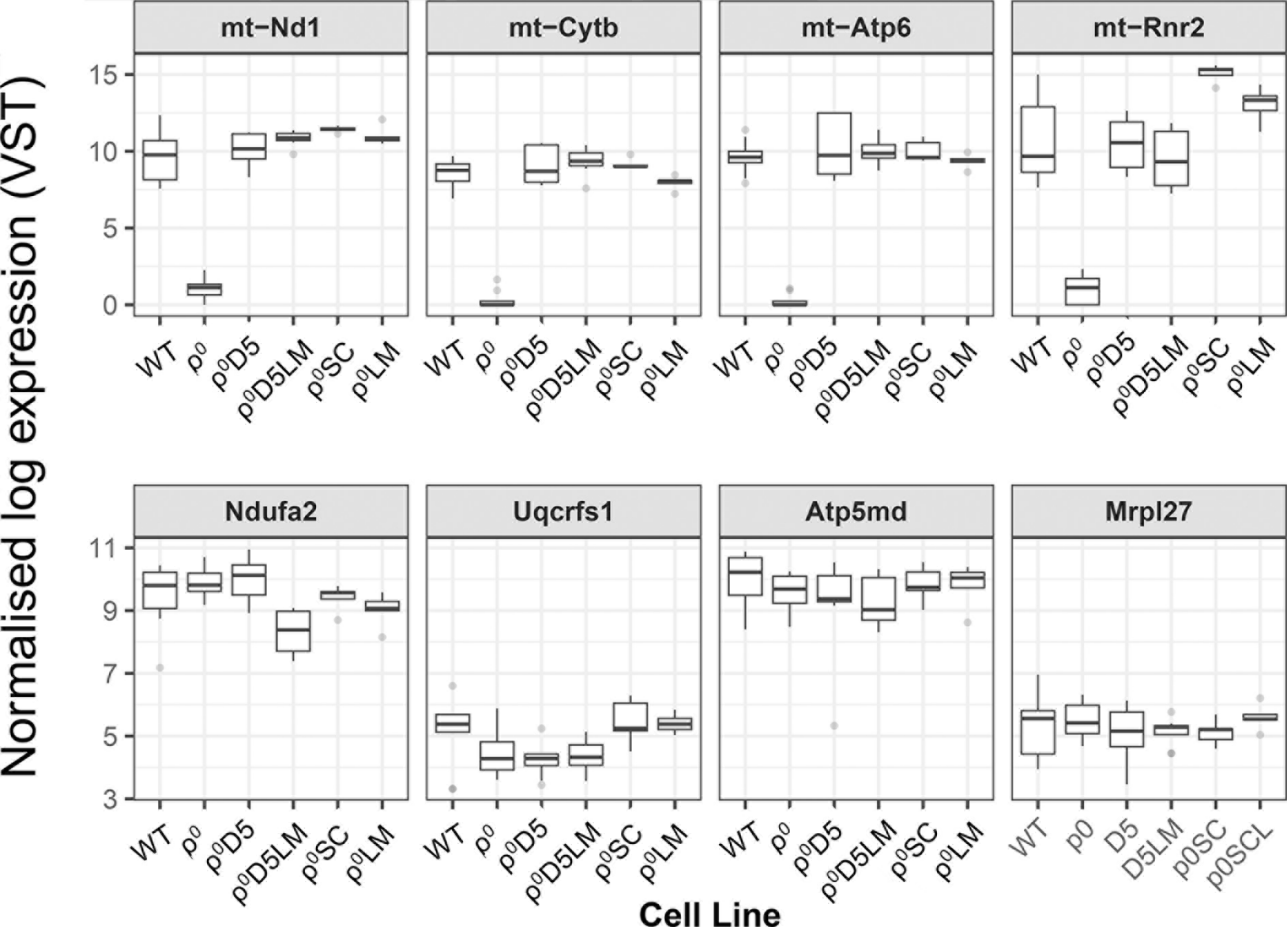
MinION nanopore RNA sequence analysis. Box plots comparing transcription of representative mitochondrially-encoded and nuclear-encoded genes between WT (n=10), ρ^0^ (n=8), ρ^0^D5 (n=8), ρ^0^D5LM (n=8), ρ^0^SC (n=5) and ρ^0^LM (n=5) cell lines. Top four squares: mitochondrially encoded genes (m-Nd1: RC I; mt-Cytb: RC III; mt-ATP6: RC V and mt-Rnr2: 16S-mtRNA. Bottom four squares: nuclear encoded genes (Ndufa2: RC I; Uqcrfs1: RC III; Atp5md: RC V and Mrp27: mitochondrial ribosomal protein 27). Boxes represent median and lower/upper quartiles, with whiskers representing minimum and maximum values. Outliers (greater than 1.5 times the inter-quartile range from the closest quartile) are plotted separately as individual points.

Next, we explored whether or not low mtDNA transcription levels could explain the inability of ρ^0^D5LM cells to increase OCR. **Figure 5** shows mtDNA transcription levels, using MinION nanopore RNA sequencing, for the mitochondrially-encoded genes m-Nd1 (RCI), mt-Cytb (RCIII), mt-Atp6 (RCV) and mt-Rnr2 (16S-RNA) (top 4 squares in **Figure 5**) as well as nuclear-encoded genes of the same respiratory complexes and mitochondrial ribosomal protein 27 (bottom 4 squares in **Figure 5**) for the different cell lines. mtDNA transcription was similar between WT, ρ^0^D5 and ρ^0^D5LM cells, ρ^0^SC and ρ^0^LM cells with mtDNA expression close to zero for ρ^0^ cells. There was also little difference in transcription of nuclear-encoded respiratory subunits between any of the cell types, consistent with previous research (24).

In summary, low respiratory capacity was not linked to poor mtDNA transcription.

**Figure 6:**
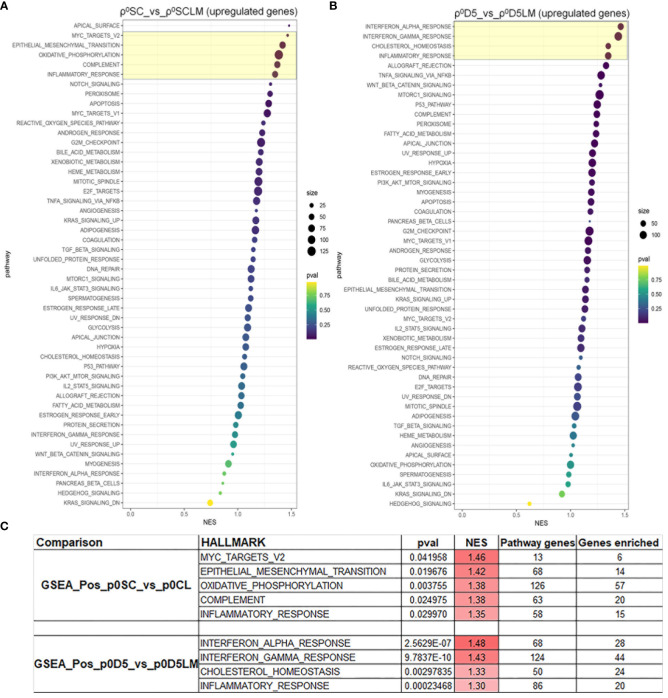
MinION GSEA analysis. Gene set enrichment analysis of upregulated pathways in ρ^0^SC vs ρ^0^LM **(A)** and ρ^0^D5 vs ρ^0^D5LM **(B)**. Pathways highlighted in yellow boxes have an NES of >1.3 and at least 20% of pathway genes enriched. **(C)** Details of the most highly enriched pathways.

Because both cell lines derived from lung metastases could not generate lung metastases themselves, we performed a gene set enrichment analysis (GSEA) of LM cell lines against their parental cell lines for the 50 hallmark gene sets (**Supplementary File 1**) (25). **Figures 6A, B** show the GSEA analysis for both cell sets; **Figure 6C** displays the pathways with a normalised enrichment score (NES) > 1.3 and at least 20% of genes of the set enriched. Only inflammation was enriched in both primary cell lines, suggesting that different factors may affect metastatic progression in the two primary cell lines.

## Discussion

We have established OXPHOS as an absolute requirement for tumorigenesis in an orthotopic mouse breast cancer model. This contradicts previous results that ATP5B-KO cells were able to establish primary tumours (12). Although we initially validated these findings in ATP5B-KO3 cells, sporadic expression of ATP5B protein, verified by fluorescence confocal microscopy, led us to generate a more stable ATP5B-KO3.1 cell line. This ATP5B-KO3.1 cell line was unable to produce primary tumours in 17 out of 18 mice or following seeding in the lungs by tail vein injection. Furthermore, several tumour cells isolated from the only primary tumour, were ATP5B positive, suggesting that tumour formation in that mouse was likely caused by a gain in ATP5B gene expression over time. It is possible that the ATP5B KO cells used previously (12) may have been unstable knockout cell lines. The absence of ATP5B protein by western blotting in small tumours of the ATP5B-KO7 cell line at early passages and in tumours taken 20 days after injection may not have reflected increasing levels of ATP5B protein in larger tumours at later time points (12).

The low basal oxygen consumption rate of the ATP5B-KO3.1 cells, similar to that of ρ^0^D5 cells, was not unexpected as these cells lack ATP synthase activity, leaving the proton motive force exclusively for importing nuclear-encoded mitochondrial proteins, calcium influx etc. Of note, tumour cells with OXPHOS levels as low as 3% of parental 4T1 cells (ρ^0^D5) could generate lung metastases (ρ^0^D5LM) and were not auxotrophic for uridine and pyruvate. Surprisingly, ρ^0^D5LM cells had the highest mtDNA levels of all cell lines tested but these cells showed no improvement in their respiratory capacity. The higher mtDNA content may have been caused by uptake of mtDNA by 4T1ρ^0^SC-D5 cells or by the mtDNA-containing ρ^0^D5 cells getting to and expanding in the lungs, or a combination of both. The lack of restoration of respiratory capacity after an increase in mtDNA content in ρ^0^D5LM cells might be explained by the fact that these cells did not need to increase their respiratory capacity in order to generate primary tumours and metastases. This phenomenon of not utilizing additional mitochondria obtained through intercellular mitochondrial transfer unless in metabolic crisis has been described recently (26).

MinION nanopore RNA sequence analysis showed no differences in mRNA levels between WT, ρ^0^D5 and ρ^0^D5LM with respect to mitochondrially encoded Nd1, Cytb and ATP6 genes, even though the mtDNA content differed significantly. This suggests that mtDNA levels have no proportional relationship with transcript levels. This discrepancy between mtDNA abundance, mtDNA transcription and respiratory competence was puzzling and at odds with previous mtDNA transcription results (12), that reported that mRNA levels of these genes in ρ^0^D5 cells were only 5% of those of WT cells. They further showed that ρ^0^D5 cells do not contain any fully assembled respiratory subunits, using blue native gel electrophoresis (12). This could explain the low OCR of both ρ^0^D5 by both our groups and potentially that of ρ^0^D5LM cells as well. We previously showed that ρ^0^SC had more than 3 times the amount of mtDNA compared with WT cells using qPCR (11). The ρ^0^SC cells were fully respiratory competent and formed lung metastases in all instances.

With respect to metastatic ability, the OXPHOS-poor ρ^0^D5 cells generated metastases, suggesting that low OCR and OXPHOS do not affect the metastatic capacity of 4T1 cells. The first step in the metastatic process is epithelial to mesenchymal transition (EMT), which was strongly enriched in ρ^0^SC cells compared to ρ^0^LM cells. Three additional enriched pathways were associated with EMT. Chronic inflammation induces EMT, and EMT further promotes immune evasion and a tumour permissive TME, characterized by low numbers of natural killer (NK) cells, dendritic cells (DCs), and cytotoxic T cells, and increased numbers of M2-polarised tumour-associated macrophages (TAMs) (27–31). The Myc family promotes EMT amongst many other pathways that lead to metastasis (27, 32, 33). Components of the complement cascade have been associated with facilitating a tumour permissive microenvironment, facilitating recruitment of tumour permissive CAFs, TAMs, TANs, MDSCs, DCregs and Tregs as well as promoting EMT (34). However, EMT is also associated with increased levels of glycolysis, decreased OXPHOS and decreased ROS levels (35, 36), suggesting that for this cell line, the increased OXPHOS pathway may endow these cells with survival advantages unrelated to their ability to metastasize.

With respect to the ρ^0^D5/ρ^0^D5LM comparison, interferon-alpha and chronic inflammation together create a tumour permissive microenvironment, not necessarily directly related to EMT, which only had an NES of 1.13 (**Supplementary Table 1**). This tumour permissive environment is further enhanced by metastatic cancer cells hijacking the interferon gamma response (37), which is also enriched in ρ^0^D5 cells. Increased cholesterol metabolism in ρ^0^D5 cells provides metastatic cancer cells with much needed cholesterol as a structural element of membranes. However, cholesterol and its derivates such as oxysterol, also promote cancer stemness, chronic inflammation and pro-tumour signalling pathways, such as MAPK and Wnt/b-catenin pathways and activation of STAT-3, which promotes EMT (38, 39).

These GSEA data show that the enriched EMT pathway is involved in the metastatic process in ρ^0^SC cells. Other pathways, that also lead to a tumour permissive microenvironment, play a larger role in ρ^0^D5 cells.

Although the reactive oxygen species (ROS) signalling pathway fell below our threshold, using our GSEA enrichment criteria, 25 genes in this pathway were enriched in both ρ^0^SC and ρ^0^D5 cells. High ROS levels that surpass the cell’s antioxidant capacity cause oxidative stress, damaged proteins, lipids and nucleic acids, leading to disease and even death. At lower levels, ROS play a vital role as secondary messengers in many pro-survival signalling pathways, including inducing EMT in various cancer cell types (40), including breast cancer (41, 42).

The majority of intracellular ROS are generated in the mitochondria during mitochondrial electron transport at complexes I and III (43). Hence, cells with low OCR likely produce fewer ROS than those with robust OCR. Although we did not measure intracellular ROS levels, because ρ^0^D5 and ρ^0^D5LM cells have similar OCR, they likely produce similar ROS levels. The fact that the ROS signalling pathway is enhanced in ρ^0^D5 cells, compared to ρ^0^D5LM cells, may be due to modifications within this pathway in the tumour microenvironment rather than the actual level of ROS generated intracellularly.

Epigenetic modifications mediated by methyltransferases, demethylases, acetyltransferases, and deacetylases affect the metastatic process of many cancers, including breast cancer (44–47). The effect of the tumour micro-environment on the epigenetics and thus metastatic ability of primary lung cancers was recently reviewed in (48). Our results suggest that the lung tumour microenvironment may also have modified those tumour cells that had established themselves in the lung, compromising their ability to metastasize when re-implanted into mice. The fact that ρ^0^D5LM cells were able to colonize the lungs after tail vein injection shows these cells can establish themselves in the lungs, but not leave the primary tumour to establish themselves in the lungs. Enrichment of the EMT pathway was much more pronounced in ρ^0^SC cells, suggesting the involvement of other signalling pathways in the metastatic process, particularly in the ρ^0^D5 cell line. It is unclear if the difference in OXPHOS levels has affected the enrichment of the EMT pathways in these two cell lines.

The fact that ρ^0^D5 cells with residual OXPHOS levels (3% of the level in parental cells) were able to generate primary tumours and metastases and the OXPHOS-null ATP5B-KO3.1 cells were not able to even generate primary tumours, shows that low levels of OXPHOS are required for tumorigenesis. Interestingly, these ATP5B-KO3.1 cells were also unable to establish lung tumours by tail vein injection. The importance of OXPHOS for cancer cells underpins various anti-cancer strategies; and our results suggest that these treatment options may not be effective in this scenario.

Previous research has shown that residual levels of OXPHOS are sufficient for generating primary 4T1 subcutaneous tumours in mice (11–13). In this manuscript we have shown that this is also the case for both primary orthotopic tumours as well as for lung metastases, obtained from these. However, the level of OXPHOS did not affect the ability to form lung metastasis from cells generated in the lung, with both poor OXPHOS-exhibiting ρ^0^D5LM cells and ρ^0^LM cells that do exhibit robust OXPHOS both unable to form lung metastases when injected orthotopically. These same cells were able to form lung lesions when injected into the tail vein, showing that the orthotopic tumours derived from robust OXPHOS ρ^0^LM cells did not disseminate to the lungs. This was supported by a downregulation in the EMT and EMT-related pathways in cells originating from the lungs, which was stronger in ρ^0^SC than in ρ^0^D5 cells. These results suggest that the lung microenvironment rather than energy metabolism *per se* constrains the metastatic potential of cells generated from lung metastases in the 4T1 model.

## Data availability statement

The original contributions presented in the study are included in the article/**Supplementary Material**, further inquiries can be directed to the corresponding author/s.

## Ethics statement

The animal study was approved by Victoria University Animal Ethics Committee. The study was conducted in accordance with the local legislation and institutional requirements. We did not use animals or humans for this study.

## Author contributions

PH: Conceptualization, Formal analysis, Funding acquisition, Investigation, Methodology, Supervision, Writing – original draft, Writing – review & editing, Project administration, Software, Visualization. GC: Investigation, Methodology, Project administration, Resources, Writing – review & editing, Software, Visualization. DL: Investigation, Methodology, Resources, Writing – review & editing, Software. DE: Data curation, Formal analysis, Investigation, Methodology, Software, Validation, Visualization, Writing – review & editing. AS: Formal analysis, Investigation, Methodology, Software, Visualization, Writing – review & editing. AW: Investigation, Methodology, Software, Visualization, Writing – review & editing. CG: Investigation, Methodology, Writing – review & editing. DO’S: Methodology, Supervision, Writing – review & editing. JN: Conceptualization, Writing – review & editing. MM: Methodology, Supervision, Writing – review & editing. MB: Conceptualization, Funding acquisition, Project administration, Supervision, Writing – review & editing.

## Supplementary material

Supplementary Figure 1 ATP5B KO cells have an unstable genotype. ATP5B protein expression in an early culture of ATP5B-KO3 **(A)** and the same culture 4 weeks later **(B)** and in cells isolated from the only primary ATP5B-KO3.1 tumor (n=18); **(C)** Seahorse profiles from WT and ATP5B-KO3 (Early: January 2021) and Late (June 2021); **(D)** Seahorse profiles of WT, ATP5B-KO3 cultured in oligomycin for 3 weeks and then 2 weeks after removal of oligomycin; **(E)** Nuclear *in situ* hybridization using the ATP5B/Cen10 FISH probes showing diploidy and triploidy of the 4T1 cell lines; **(F)** Example of few ATP5B positive cells generated from the primary tumor of 1 mouse (out of 18) injected with ATP5B-KO3.1; **(G)** Cytogenetic annotations for the different cell lines. Seahorse data are an average and SEM of 2 biological replicates.

Supplementary Figure 2 Image analysis diagram highlighting each of the steps for mtDNA nucleoid quantification per cell. **(A)** original image stained with MitoTracker CMXROS (Magenta), SYBR Gold (Green) and Hoechst (Blue); **(B)** Nuclear segmentation; **(C)** Cell segmentation based on MitoTracker staining; **(D)** SYBR Gold segmentation; **(E)** Nuclei are subtracted from the cell segmentation to remove SYBR gold signal from the nuclei; **(F)** The number of SYBR Gold staining particles inside each segmented cell minus SYBR Gold staining particles underneath or on top of the nucleus can be identified and counted. Scale bar 50 µm.
