# Supplementary figures and images for: Residual OXPHOS is required to drive primary and metastatic lung tumours in an orthotopic breast cancer model
Source: Front Oncol. 2024 May 1;14:1362786. doi: 10.3389/fonc.2024.1362786 (PMC11094293; doi:10.3389/fonc.2024.1362786)

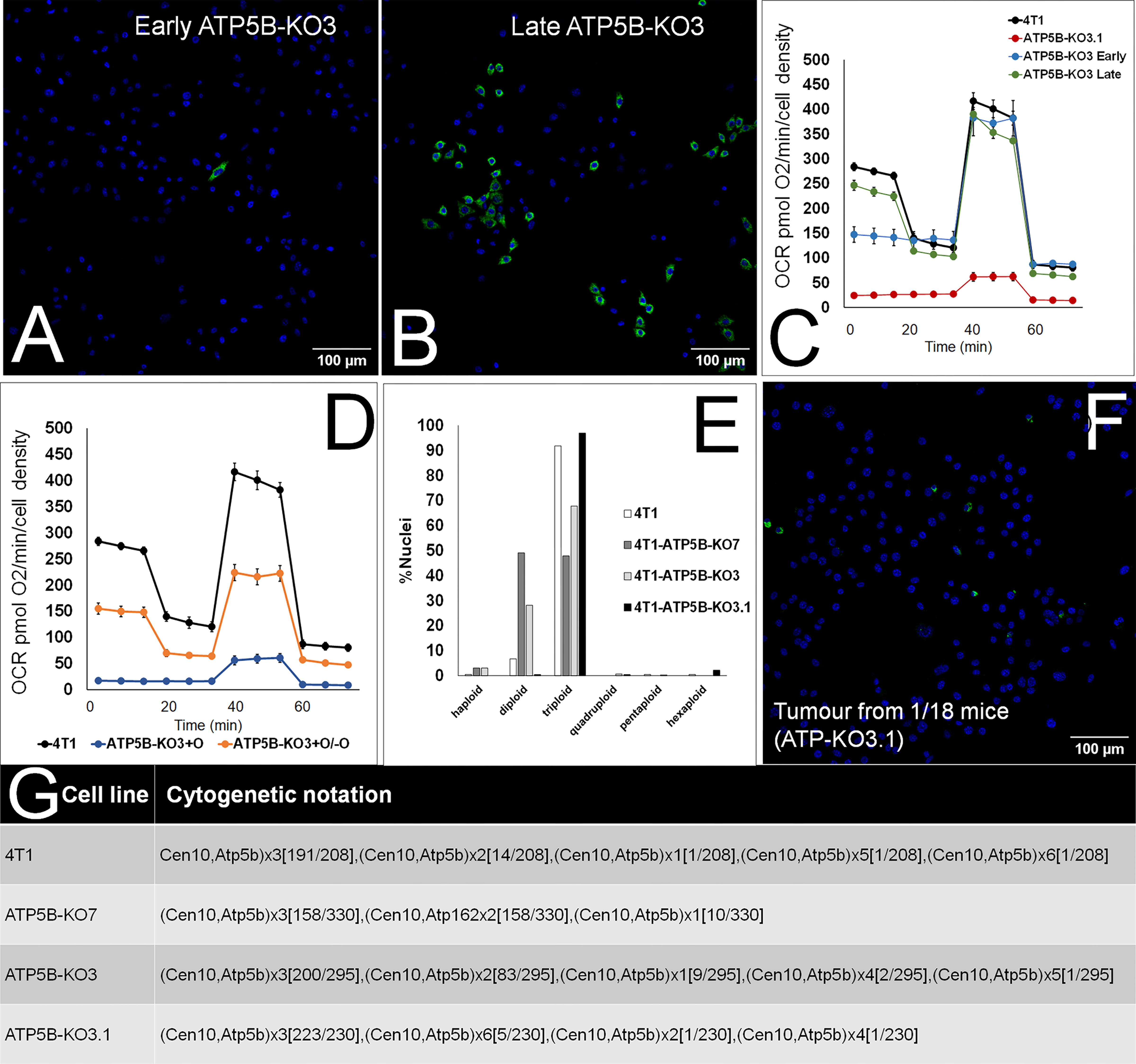

Supplement: Supplementary Figure 1 — ATP5B KO cells have an unstable genotype. ATP5B protein expression in an early culture of ATP5B-KO3 (A) and the same culture 4 weeks later (B) and in cells isolated from the only primary ATP5B-KO3.1 tumor (n=18); (C) Seahorse profiles from WT and ATP5B-KO3 (Early: January 2021) and Late (June 2021); (D) Seahorse profiles of WT, ATP5B-KO3 cultured in oligomycin for 3 weeks and then 2 weeks after removal of oligomycin; (E) Nuclear in situ hybridization using the ATP5B/Cen10 FISH probes showing diploidy and triploidy of the 4T1 cell lines; (F) Example of few ATP5B positive cells generated from the primary tumor of 1 mouse (out of 18) injected with ATP5B-KO3.1; (G) Cytogenetic annotations for the different cell lines. Seahorse data are an average and SEM of 2 biological replicates. [file Image_1.tif]

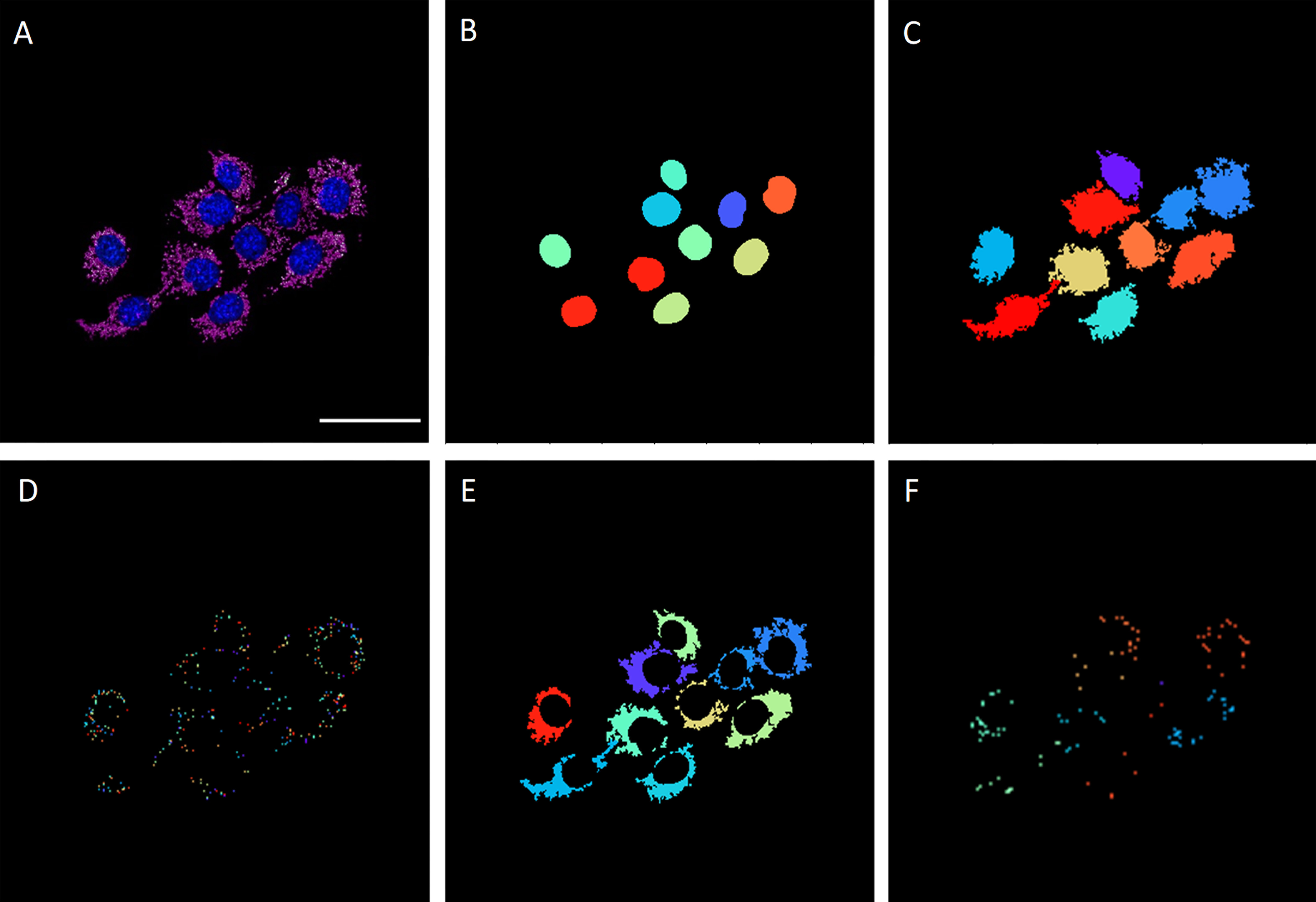

Supplement: Supplementary Figure 2 — Image analysis diagram highlighting each of the steps for mtDNA nucleoid quantification per cell. (A) original image stained with MitoTracker CMXROS (Magenta), SYBR Gold (Green) and Hoechst (Blue); (B) Nuclear segmentation; (C) Cell segmentation based on MitoTracker staining; (D) SYBR Gold segmentation; (E) Nuclei are subtracted from the cell segmentation to remove SYBR gold signal from the nuclei; (F) The number of SYBR Gold staining particles inside each segmented cell minus SYBR Gold staining particles underneath or on top of the nucleus can be identified and counted. Scale bar 50 µm. [file Image_2.tif]
